# Electron Beam Irradiation Modulates the Multiscale Structure and Physicochemical Properties of Wheat Starch in Dough Systems

**DOI:** 10.3390/foods15061005

**Published:** 2026-03-12

**Authors:** Yaru Yuan, Peishan Liu, Yanyan Zhang, Yingying Zhang, Mengkun Song, Hongwei Wang, Huishan Shen, Hua Zhang, Xingli Liu

**Affiliations:** 1 College of Food and Bioengineering, Zhengzhou University of Light Industry, Zhengzhou 450001, China; yuan1024851842@163.com (Y.Y.); wangmoran369@163.com (P.L.); zhangyanyan@zzuli.edu.cn (Y.Z.); zhangyingying@zzuli.edu.cn (Y.Z.); 2025012@zzuli.edu.cn (M.S.); hwwang@zzuli.edu.cn (H.W.); shen1685778117@163.com (H.S.); 2 National & Local Joint Engineering Research Center of Cereal-Based Foods (Henan), Zhengzhou 450001, China; 3 Henan Province Major University-Enterprise Joint R&D Centre for Quick-Frozen Flour and Prepared Foods, Zhengzhou University of Light Industry, Zhengzhou 450001, China

**Keywords:** dough quality, electron beam irradiation, molecular structure, wheat flour, wheat starch

## Abstract

Wheat is rich in carbohydrates and proteins but is susceptible to pest infestation and microbial contamination during storage. Owing to itself high efficiency, energy savings, and lack of chemical residues, electron beam irradiation (EBI) has been widely applied for disinfesting and sterilizing cereals and has been shown to influence dough quality. Notably, starch is present within complex wheat flour systems during processing, and its irradiation response may differ from that of purified systems. In this study, the effects of different EBI doses (0, 3, 6, 9 and 12 kGy) on the multiscale structure and physicochemical properties of wheat starch isolated from irradiated dough were systematically investigated, and key analytical techniques such as Fourier transform infrared spectroscopy (FTIR), X-ray diffraction (XRD), and rheological analysis were employed to elucidate the mechanisms underlying its impact on the dough thermomechanical behavior of dough. The results demonstrated that EBI weakened gluten–starch interactions and disrupted gluten network the continuity and compactness of the gluten network, resulting in significant dough farinography and pasting property changes. Compared with those of the control group, the dough development and stability time of the 12 kGy sample decreased from 3.920 and 6.465 to 0.970 and 1.290, respectively (*p* < 0.05). Moreover, irradiation induced cracks on the starch surface, reduced its molecular weight, and disrupted its crystallinity and short-range order. These changes resulted in decreases in the thermal stability level and swelling capacity of starch, while increasing its solubility. A correlation analysis revealed that the starch chain length distribution, molecular weight, molecular order, and pasting properties are key determinants of EBI-induced dough quality changes. This study provides theoretical insights into the applicability of EBI in the context of wheat flour storage and quality modulation.

## 1. Introduction

Wheat flour should be a major raw material in the global food-processing industry because of its stable supply, high processing adaptability, and diverse functional properties, and it is widely used in the production of bread, noodles, steamed bread, and various baked products [1,2,3]. However, wheat is rich in carbohydrates and proteins, which establish a favorable nutritional environment for the growth and proliferation of pests and microorganisms, making it highly susceptible to pest infestations and microbial contamination during storage [4]. These situations result in reduced product quality and economic losses [5]. Therefore, the development of safe and efficient storage and preservation technologies that are capable of suppressing pests and microbial spoilage while minimizing post-harvest losses has become a critical issue for ensuring food security.

Electron beam irradiation (EBI) has emerged as a green and efficient grain preservation technology owing to its high processing efficiency, precise dose controllability, and avoidance of chemical additives [6,7]. Consequently, it has shown considerable potential for ensuring food safety and regulating the quality of grains and raw food materials. Extensive studies have explored the effects of irradiation on the storage stability and quality attributes of wheat flour, primarily focusing on the inactivation of pathogenic and spoilage microorganisms [8,,9], and the inhibition of germination [10]. Moreover, attention has been given to the effects of irradiation on the quality of macroscopic processing schemes, such as improving the specific volume of the bread [11] or modifying the pasting and thermal properties of wheat starch [12]. However, compared with these macroscopic evaluations, systematic investigations into the structural alterations exhibited by the key internal components of wheat flour due to irradiation remain limited. Previous studies have demonstrated that irradiation can induce molecular chain scission, disrupt crystalline domains, and reduce the short-range order, thereby markedly altering the physicochemical properties of starch [13,14]. Notably, under practical processing conditions, starch does not exist as an isolated component but rather as part of a complex wheat flour system, and its structural response to irradiation may differ from those of purified starch systems. Accordingly, a systematic investigation of the irradiation-induced modifications exhibited by the multilevel structure and physicochemical properties of isolated wheat starch is essential to gain deeper mechanistic insights into the ways in which EBI modulates the quality of wheat flour.

Starch is the predominant component of wheat flour, accounting for approximately 70–75% of its total mass, and it plays a pivotal structural and functional role in the wheat flour system [15]. The multiscale structural characteristics and physicochemical properties of starch have considerable impacts on the formation process and stability of the dough system, thereby governing the quality attributes of final wheat-based products [12]. During processing, the water absorption, swelling, and gelatinization behaviors of starch provide crucial physical support for the development of the dough structure, serving as a fundamental basis for regulating the structure and performance of the dough [16,17]. Consequently, manipulating the multilevel structure of starch to achieve targeted wheat flour functionality modulation has attracted increasing interest in the fields of cereal science and food processing [18].

Therefore, wheat flour (durum wheat) was treated with different EBI doses in this study, and the thermomechanical behavior of dough made from the irradiated wheat flour was systematically evaluated. Moreover, the multilevel structural characteristics (the morphology of starch granules, chain length distribution, crystalline structure, molecular weight, and short-range order) and physicochemical properties of isolated wheat starch were comprehensively characterized. On this basis, the intrinsic relationships between starch multiscale structural starch alterations and the associated dough quality deteriorations were further elucidated. This study clarified the mechanisms underlying the EBI-induced regulation of wheat flour quality from a structure–property–function perspective and provided a basis for the rational use of irradiation in the quality control schemes for wheat flour and related products.

## 2. Materials and Methods

### 2.1. Materials

Eight-star flour was obtained from Wudeli Flour Group Co., Ltd. (Hebei, China). The starch and protein contents of the wheat flour were 73.0% and 9.6%, respectively. Dimethyl sulfoxide (DMSO) was purchased from Anpu Experimental Technology Co., Ltd. (CNW, Shanghai, China). FITC, rhodamine B, NaOH and KBr were purchased from Aladdin Biochemical Technology Co., Ltd. (Shanghai, China). LiBr and NaAc were obtained from Sigma Chemicals (St. Louis, MO, USA). All the chemicals and reagents applied in this work were of high-purity analytical grade.

### 2.2. EBI of Wheat Flour and Dough Preparation

EBI was applied to the wheat flour at Zhengzhou Hongyuan Bioengineering Co., Ltd. (Zhengzhou, China). Wheat flour samples (500 g) were sealed in polythene bags (240 mm × 340 mm) and evenly placed on a conveyor belt for irradiation. The target irradiation doses were set to 3, 6, 9, and 12 kGy, with a dose rate of 1 kGy/h. Following the irradiation step, the dough was prepared by mixing wheat flour samples treated with different doses of deionized water at 50% (*w*/*w*, based on the flour weight) in a mixer for 10 min. Then, the dough was removed, covered with plastic wrap to prevent the loss of moisture, and rested in a constant-temperature chamber (HWS-080; Jinghong Experimental Equipment Co., Ltd., Shanghai, China) at 30 °C for 30 min prior to conducting the subsequent analyses. The samples were separately designated as nonirradiated samples (control, 0 kGy) and irradiated samples.

### 2.3. Thermomechanical and Microstructural Properties of Wheat Flour Dough

#### 2.3.1. Thermomechanical Behavior of Irradiated Dough

The thermomechanical behavior of the samples were measured using a Mixolab analyzer (Chopin Technologies, Villeneuve-la-Garenne, France) by following the manufacturer’s standard protocol [19]. The dough was prepared at a target torque of 1.1 ± 0.05 N·m. The degrees of water absorption, development, stability, and softening degree were recorded and analyzed.

#### 2.3.2. Microstructure of the Irradiated Dough

##### Confocal Laser Scanning Microscopy (CLSM)

Fresh dough with the appropriate size was removed from the center, and the samples were fixed in 2.5% (*v*/*v*) glutaraldehyde solution at 4 °C for 12 h. After the fixation process was completed, the dough was prepared as 50 μm slices using a freezing microtome, stained with a mixed dye solution consisting of FITC and rhodamine B in the dark, and then observed under an FV3000 laser confocal microscope (Olympus, Tokyo, Japan). The excitation wavelength of FITC was 488 nm, and that of rhodamine B was 561 nm.

##### Scanning Electron Microscopy (SEM)

The freeze-dried dough samples were placed on the sample stage with conductive silicone tape for analysis purposes. The microscopic morphology was observed using a JEM-6490 LV field emission SEM system (ZEISS, Oberkochen, Germany) at 3 kV and a magnification of 1000× after applying a gold spraying treatment.

### 2.4. Isolation of Starch from Wheat Dough

Starch was extracted from the wheat flour following a previously reported method with minor modifications [20]. The dough was dispersed in deionized water and manually washed for 10 min. Insoluble residues were removed from the starch slurry by passage through a 100-mesh sieve, followed by the addition of 0.1% (*w*/*v*) NaOH with continuous stirring. Afterwards, the slurry was centrifuged at 4000 rpm for 10 min. After undergoing centrifugation, the upper pigmented layer was carefully removed, and the lower white starch fraction was collected. The washing and centrifugation steps were repeated until the starch was sufficiently purified. The pH of the suspension was subsequently adjusted to 7.0 with 1 M of HCl. The samples were dried at 45 °C.

### 2.5. Multiscale Structure of the Isolated Wheat Starch

#### 2.5.1. SEM

The freeze-dried starch samples were gently fractured to avoid causes structural damage. Samples with smooth and uniform surfaces were placed on sample plates, sprayed with gold, and observed at 3 kV under 1000× magnification.

#### 2.5.2. Chain Length Distribution (CLD)

The CLD was analyzed through high-performance size exclusion chromatography (HPSEC) [21]. The purified starch (10 mg) was suspended in deionized water (5 mL) and gelatinized in a boiling water bath for 60 min. After being cooled, the mixture was treated with 50 μL of NaAC buffer (0.6 M, pH 4.4), 10 μL of NaN_3_ solution (2%, *w*/*v*), and 10 μL of isoamylase (1400 U). Afterwards, it was incubated at 37 °C for 24 h to debranch the starch molecules. Sodium borohydride solution (0.5%, *w*/*v*) was subsequently added to the mixture, followed by vortexing, and the mixture was allowed to react for 20 h to terminate the reaction. An aliquot of 600 μL was evaporated under nitrogen at the ambient temperature. The residue was solubilized in 30 μL of 1 M NaOH for 60 min, diluted with 570 μL of deionized water, and centrifuged at 12,000 rpm for 5 min. The supernatant was subsequently harvested for analysis purposes. The CLD of the starch was analyzed using a Dionex™ CarboPac™ PA200 column (10 μm; Thermo Fisher Scientific, Waltham, MA, USA). The other conditions included a column temperature of 30 °C, an injection volume of 5 μL, and a flow rate of 0.4 mL/min, and the mobile phases consisted of 0.2 M NaOH and 0.2 M NaOH–NaAC.

#### 2.5.3. Molecular Weight (Mw)

The Mw was analyzed by a high-performance size exclusion-based chromatography system coupled with multiangle laser light scattering and a refractive index detector (HPSEC–MALLS–RID). Starch dissolved in DMSO (containing LiBr) to prepare a sample solution (0.1%, *w*/*v*), which was used as the mobile phase. Afterwards, it was heated at 80 °C for 3 h to ensure the complete dissolution of the starch molecules. After being cooled, the solution was filtered through a 0.45 μm membrane filter and injected into an OHpak SB-806HQ series size-exclusion column (8.0 mm × 300 mm) installed on an Ultimate 3000 HPLC system (Thermo Fisher Scientific, Waltham, MA, USA). The other conditions included a column temperature of 60 °C and a flow rate of 0.3 mL/min.

#### 2.5.4. Crystalline Structure

X-ray diffraction (XRD) was employed to analyze the crystalline structure of the starch using a SmartLab SE diffractometer (Rigaku, Tokyo, Japan) at 40 kV and 30 mA. The experimental conditions were as follows: a step size of 0.02°, a scanning rate of 5°/min, and a scanning range of 5–50° (2θ). Relative crystallinity (RC) values were calculated by separating crystalline and amorphous regions using peak deconvolution.

#### 2.5.5. Short-Range Ordered Structure

##### Fourier Transform Infrared (FT-IR) Spectroscopy

A Vertex 70 FT-IR spectrometer (Bruker, Karlsruhe, Germany) was used to evaluate the short-range order of the starch. Pre-dried KBr was thoroughly blended with the samples at a mass ratio of 100:1 (*w*/*w*), finely ground, and compressed into transparent pellets. The spectra ranged from 4000 cm^−1^ to 400 cm^−1^. The spectral data were processed by conducting baseline correction and deconvolution using OMNIC 8.0, followed by peak fitting with the PeakFit 4.12 software.

##### Raman Spectroscopy

A portable Raman spectrometer (BWS 465-785S, Microtrac Inc., Largo, FL, USA) was used to acquire eight cumulative scans at a 785 nm excitation wavelength. The laser power lever was set to 100%, and the scanning range extended from 4000–320 cm^−1^. The full width at half maximum (FWHM) of the 480 cm^−1^ absorption band was determined to evaluate the short-range order of the starch.

### 2.6. Physicochemical Properties of the Isolated Wheat Starch

#### 2.6.1. Amylose Content (AC)

The AC determination procedure was performed according to GB/T 15683-2008 [22]. Briefly, the starch samples were completely gelatinized in an alkaline solution, followed by complexation with an iodine–potassium iodide solution. After applying color development, the absorbance lever was recorded at the characteristic wavelength using a UV-Vis spectrophotometer (T6, Puxi General Instrument Co., Ltd., Beijing, China). The AC was quantified on the basis of a calibration curve established from standard amylose.

#### 2.6.2. Solubility and Swelling Power

Starch powder (2 g, with the weight of dry starch was recorded as M) was dispersed in distilled water to prepare a 2% (*w*/*w*, dry basis) sample solution and heated at 85 °C for 30 min. The solution was centrifuged at 3500 rpm for 20 min after cooling. The precipitate mass was measured defined as M_1_. The supernatant was transferred to an aluminum dish and dried at 105 °C to produce a constant weight, and the resulting dry mass was recorded as M_2_. The starch solubility and swelling characteristics were calculated as follows:

(1) $$Solubility\;(\%)=\frac{M_{2}}{M}\times 100\%$$

(2) $$Swellingpower\;(g/g)=\frac{M_{1}}{M-M_{2}}\times 100\%$$

#### 2.6.3. Thermal Properties

The thermal properties of the starch were determined using differential scanning calorimetry (DSC Q20, TA Instruments, New Castle, DE, USA). The starch samples were mixed with deionized water at a ratio of 1:3 (*w*/*w*) and placed in a sealed aluminum pan. It was then left at 4 °C overnight to fully equilibrate. The empty sealed pan served as a control. The samples were heated from 20 to 120 °C at a rate of 10 °C/min.

#### 2.6.4. Pasting Properties

The pasting properties of the starch were analyzed using the Rapid Visco Analyzer (RVA, Perten Instruments, Stockholm, Sweden). Briefly, 3.0 g (dry basis) of starch was uniformly dispersed in 25 g of distilled water in an RVA canister and thoroughly mixed. Afterwards, the sample was held at 50 °C with a stirring speed of 960 rpm for 1 min, heated to 95 °C at 12 °C/min with a stirring speed of 160 rpm for 2.5 min, cooled to 50 °C at 12 °C/min and finally held at that temperature holding for 2 min.

### 2.7. Statistical Analysis

All the experiments were repeated three times. The results are expressed as mean ± standard deviation. The statistical analysis was performed using SPSS 27 software, and significant differences were evaluated at *p* < 0.05.

## 3. Results and Discussion

### 3.1. Effect of EBI on the Thermomechanical Behavior of Dough

Thermomechanical properties refer to the mechanical responses and structural evolution trends of flour systems under the combined effects of temperature variations and mechanical forces (such as shearing, oscillation, or compression), reflecting their structural stability and functional performance during processing and thermal treatment [19]. Compared with that of the nonirradiated samples, the water absorption rate of the irradiated dough slightly increased, whereas the development time generally tended to decrease. As the irradiation dose increased, the stability time significantly decreased (*p* < 0.05), whereas the degree of weakening significantly increased with increasing dose (*p* < 0.05) (Table 1). This effect was likely caused by irradiation-induced degradation and structural rearrangement processes, which increased the exposure of hydrophilic groups and increased the water binding capacity of the dough. Moreover, gluten protein chain scission reduced the continuity and stability of the protein network [23], making it difficult for dough to form and maintain a stable structure, thereby shortening its development and stability times. The shear resistance of the gluten network further decreased, as evidenced by a decrease in the gluten strength of the dough and a significant increase in the degree of weakening (*p* < 0.05) [11].

The peak viscosity (PV), trough viscosity (TV), final viscosity (FV), and setback value (SB) can be used to reflect the degree of binding between flour particles and water molecules at a specific temperature [24]. The PV, TV, FV, and SB of the irradiated dough were decreased significantly compared with those of the nonirradiated dough (*p* < 0.05), indicating the typical characteristics of irradiation-modified samples (Table 1). This effect can be primarily attributed to the electron beam irradiation-induced scission of starch chains and the disruption of the crystalline structure, which reduced the structural stability and swelling capacity under heating and shear, thereby leading to a marked reduction in the pasting parameters [25,26].

### 3.2. Effect of EBI on the Microstructure of Dough

**Figure 1:**
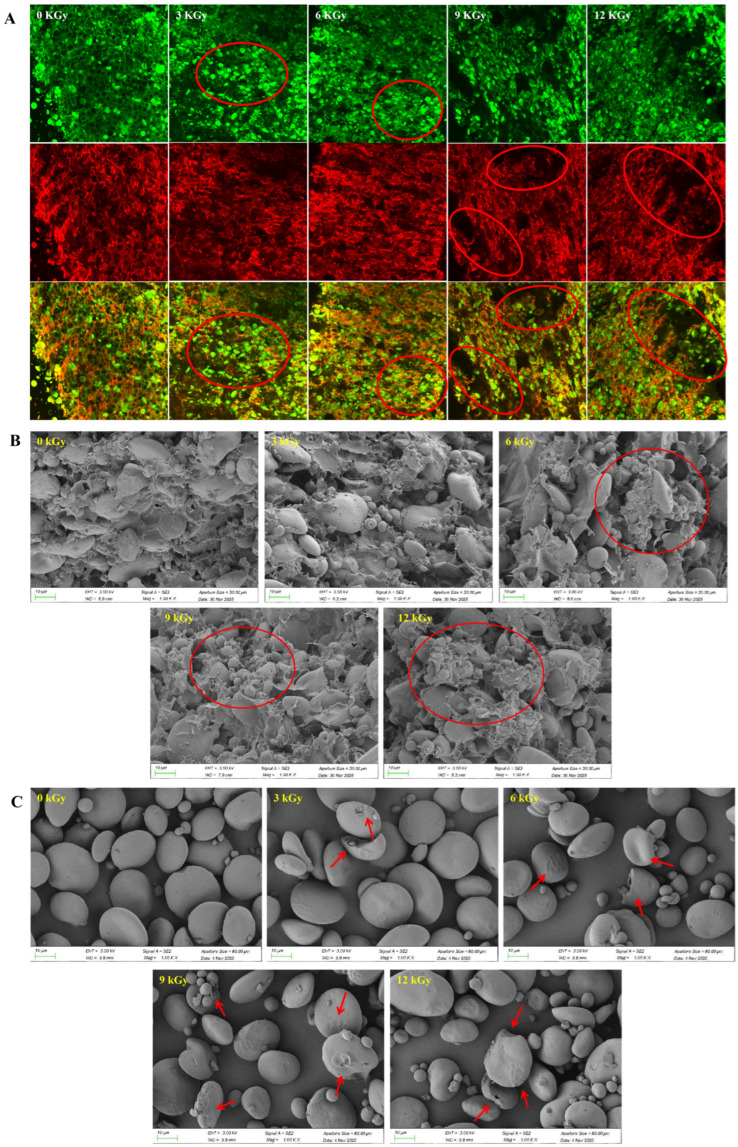
Effect of electron beam irradiation on the microstructure of dough and starch granules. (**A**) CLSM images of dough. (**B**) SEM images of dough. (**C**) SEM images of starch granules.

The CLSM images of the dough are shown in Figure 1A. In the nonirradiated dough, the gluten proteins formed a continuous and compact network in which starch granules were uniformly distributed and effectively embedded, indicating strong gluten–starch interactions. The continuity and integrity of the gluten network were progressively weakened with increasing dose, and this was accompanied by a marked reductions in protein–starch cross-linking and cooperative interactions, resulting in an increased exposure for the starch [27]. Similarly, Shen et al. reported that EBI induced similar effects on the CLSM images of wheat starch [12].

Consistent observations were obtained from the SEM images (Figure 1B). The nonirradiated dough exhibited a dense and intact microstructure, where the starch granules were tightly encapsulated by a thick and continuous gluten protein network, which is consistent with the results that have been reported for wheat dough [28]. The gluten protein became thinner and fragmented with increasing dose, leading to reduced network continuity and the formation of larger voids. Similarly, the starch granules became increasingly exposed, and their surfaces displayed pronounced granular features even when partially embedded, further indicating that the cross-linking and interlocking between gluten protein and starch have been significantly weakened.

Collectively, these microstructural changes demonstrated that EBI disrupted the gluten–starch network, which ultimately contributed to the deterioration of dough rheological properties. These findings were in agreement with the CLSM observations.

### 3.3. Effects of EBI on the Multiscale Structure of Starch

#### 3.3.1. SEM of Wheat Starch Granules

Overall, the structural integrity of the starch granules was not entirely destroyed by EBI (Figure 1C). In the nonirradiated samples, the starch granules exhibited smooth and compact surfaces with well-preserved structural integrity. With increasing irradiation dose, the granule surfaces progressively developed pits and wrinkles, which were accompanied by a pronounced increase in surface roughness. Under high-dose irradiation, partial granule damage was observed, along with the formation of fragmented particles possessing reduced sizes. These alterations were closely associated with the generation of high-energy free radicals during EBI, which induced the scission of starch molecular chains. The resulting degradation weakened the internal structural stability of the starch granules, thereby promoting surface erosion and localized structural failure. These results indicated that EBI could significantly affect the microscopic morphology of starch granules, and that this effect intensified with increasing dose. Earlier studies reported similar phenomena in rice and potato starch [29,30].

#### 3.3.2. The Mw and CLD of Wheat Starch

The Mw of starch significantly decreased from 85,003.53 g/mol (0 kGy) to 8734.38 g/mol (12 kGy) with increasing dose (*p* < 0.05) (Table 2). Liang et al. reported similar results for sorghum starch [31]. The underlying reason for this finding was that the free radicals generated during EBI attacked the glycosidic bonds of starch molecular chains, inducing the degree of random chain scission and the formation of abundant short-chain molecules, thereby leading to an overall decrease in the molecular weight of the starch [32].

**Figure 2:**
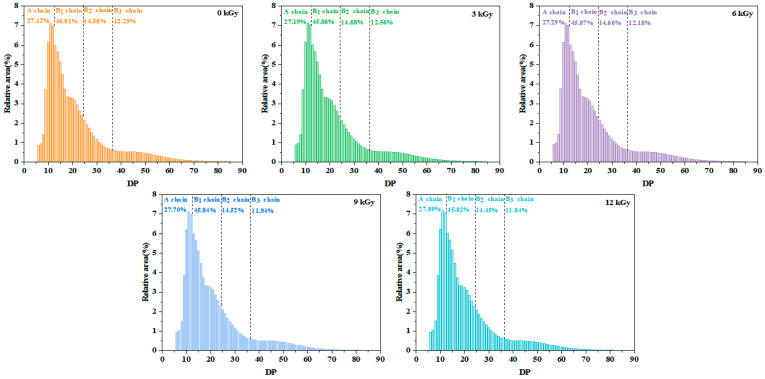
Effect of electron beam irradiation on the distribution of starch chain length.

The amylopectin chains are conventionally divided into A chains (DP 6–12), B_1_ chains (DP 13–24), B_2_ chains (DP 25–36), and B_3_ chains (DP > 36) based on the basis of their degrees of polymerization (DP). EBI led to a dose-dependent increase in the number of A chains and an overall decrease in the number of B chains (Figure 2). This occurred because the free radicals formed during irradiation disrupted the starch granule structure, causing the cleavage of the glycosidic bonds and the depolymerization of long amylopectin chains into shorter chains [33]. These results aligned with the influences of EBI on the dry-heated buckwheat starch [34].

#### 3.3.3. Crystalline Structure of Wheat Starch

**Figure 3:**
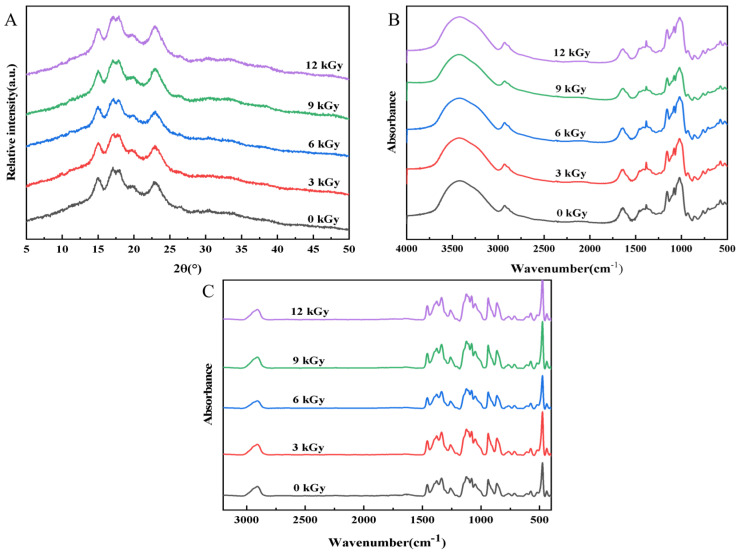
Effect of electron beam irradiation on starch construction. (**A**) XRD pattern. (**B**) FTIR spectra. (**C**) Raman spectra.

The wheat starch exhibited distinct characteristic peaks at 2θ = 15°, 17°, 18°, and 23°, indicating an A-type crystalline structure (Figure 3A). The positions of these characteristic peaks remained essentially unchanged after undergoing EBI, and no new peaks appeared nor did the existing peaks disappear, suggesting that the An-type crystalline structure of the wheat starch was preserved.

Although the crystalline type remained unchanged, EBI significantly affected the crystallinity of starch. EBI led to a significant decrease in the relative crystallinity (RC) level of the starch, which decreased in a dose-dependent manner (Table 2). Specifically, the RC significantly decreased from 31.82% (0 kGy) to 25.43% (12 kGy) (*p* < 0.05). Similar findings have been reported for oat starch and lotus seed starch [18,35]. These results indicated that EBI disrupted the crystalline regions of starch, resulting in a reduction in the level of structural order. This effect was due to EBI inducing chain scission and depolymerization through free radical reactions, which disrupted the ordered double-helix structures between amylopectin side chains and amylose. It also weakened the stability of the intramolecular hydrogen-bond network, thereby reducing the crystallinity and structural integrity of starch [36].

#### 3.3.4. FT-IR and Raman Analyses of Wheat Starch

The peak intensity ratio R_1045/1022_ is widely applied to characterize changes in the short-range order of starch, and the FWHM of the Raman band produced at 480 cm^−1^ is indicative of the regularity of starch double-helical starch structures. The combined use of FT-IR and Raman spectroscopy enables a comprehensive assessment of irradiation-induced changes in the short-range order of starch [37].

The FT-IR and Raman spectra of the nonirradiated sample and irradiated samples produced at different doses were similar, reflecting the notion that EBI did not introduce new functional groups (Figure 3B,C). The chemical composition and fundamental molecular framework of the wheat starch thus remained essentially unchanged, which was in line with previous reports by Wang et al. and Shen et al. [12,13]. Nevertheless, with increasing dose, the R_1045/1022_ of starch decreased from 0.870 to 0.805, whereas the FWHM significantly increased from 16.49 to 19.31 (*p* < 0.05) (Table 2). These results suggested that EBI induced starch degradation and markedly weakened its short-range ordered structure. Similar trends have also been reported for rice starch subjected to EBI (0 to 6 kGy) [38]. It has been suggested that the free radicals generated during irradiation trigger starch chain scission, weaken the intramolecular and intermolecular hydrogen bonds, and lead to the unwinding of double-helical structures within crystalline regions, thus reducing the short-range molecular order [39].

### 3.4. Effects of EBI on the Physicochemical Properties of Wheat Starch

#### 3.4.1. Amylose Content (AC) of Wheat Starch

The AC of nonirradiated sample was 335.30 mg/g. After applying electron beam irradiation, the AC significantly increased (*p* < 0.05) at a dose of 9 kGy, reaching a maximum of 373.31 mg/g at 9 kGy, and then slightly decreased at 12 kGy to a level close to that observed at 6 kGy, indicating a nonmonotonic trend (Table 2). This increase was likely attributed to the irradiation-induced cleavage of glycosidic bonds. The free radicals generated during EBI could promote the debranching of amylopectin and the depolymerization of long-chain amylose, which contributed to a greater fraction of shorter amylose chains, thereby increasing the AC. At higher doses (12 kGy), further chain scission may have degraded some short-chain amylose into low-molecular-weight sugars, or induced molecular rearrangement and aggregation processes, leading to a slight decrease in the AC.

However, studies have revealed that the effects of irradiation on the AC of starch derived from different sources are not consistent. While Zhao et al. observed a dose-dependent decline in the AC for acorn and pea starches under EBI [40,41], AC in potato starch increased following EBI and X-ray treatment [29]. In conclusion, the effect of irradiation treatment on the AC of starch does not follow a uniform pattern. Its change trend may be comprehensively affected by multiple factors, such as the irradiation type and dose, the starch origin, and molecular structure characteristics [40]. These contrasting outcomes reflected inherent differences in molecular architecture and irradiation conditions. At low doses, preferential cleavage of amylopectin branch chains generates shorter linear fragments that mimic amylose in iodine-binding assays, yielding an apparent AC increase. However, higher doses further degrade amylose, resulting in reduced AC. Thus, irradiation-induced changes in starch AC are non-linear and dictated by the interplay of structural characteristics and processing parameters.

#### 3.4.2. Solubility and Swelling of Wheat Starch

EBI significantly altered the solubility and swelling of isolated wheat starch. As the dose increased from 0 to 12 kGy, the degree of solubility increased markedly from 7.30% to 34.75% (*p* < 0.05), whereas the swelling power decreased significantly from 10.29% to 7.82% (*p* < 0.05) (Table 2). This significant increase in solubility was attributed mainly to the free radical-mediated scission of starch molecule chains during irradiation, which generated abundant low-molecular-weight fragments that interacted more readily with water and diffused out of the granules [42]. Similarly, researchers reported that EBI improved the cold-water solubility of chestnut starch [43].

In contrast, the reduction in swelling may have resulted from the irradiation-induced disruptions of the granule integrity level and crystalline regions, which weakened the structural framework of starch. In addition, the short-chain components produced by chain scission were less capable of forming an effective network that could support granule expansion, thereby limiting the degrees of water absorption and swelling. The influence of irradiation on the swelling power of starch is governed by several factors, such as the irradiation source, dose level, and the origin of starch. According to a report published by Smita et al., the swelling exhibited by irradiated samples was greater than that of nonirradiated samples [44]. However, the swelling of oat starch increased in a dose-dependent manner [18].

#### 3.4.3. Thermal Properties of Wheat Starch

The To, Tp, Tc and ΔH of the nonirradiated sample were 58.99 °C, 63.43 °C, 80.16 °C, and 10.94 J/g, respectively (Table 3). Compared with the nonirradiated sample, EBI caused significant and dose-dependent decreases in To, Tp, and ΔH (*p* < 0.05), whereas Tc was only slightly affected. At the highest irradiation dose of 12 kGy, To, Tp, and ΔH decreased to 57.15 °C, 62.06 °C, and 9.00 J/g, respectively. These results indicated that EBI induced starch molecular chain degradations in the starch through free radical-mediated glycosidic bond cleavage, disrupting crystalline regions and reducing the molecular order and double-helix stability level, thereby weakening the thermal stability of the crystals and facilitating gelatinization, which led to an overall decline in the thermal parameters. Similarly, previous studies on maize starch [45] and waxy maize starch [33] reported decreases in To and Tp after applying irradiation, which were ascribed to partial amylopectin depolymerization and crystalline region disruption—mechanisms fully in line with those observed here.

#### 3.4.4. Pasting Properties of Wheat Starch

**Figure 4:**
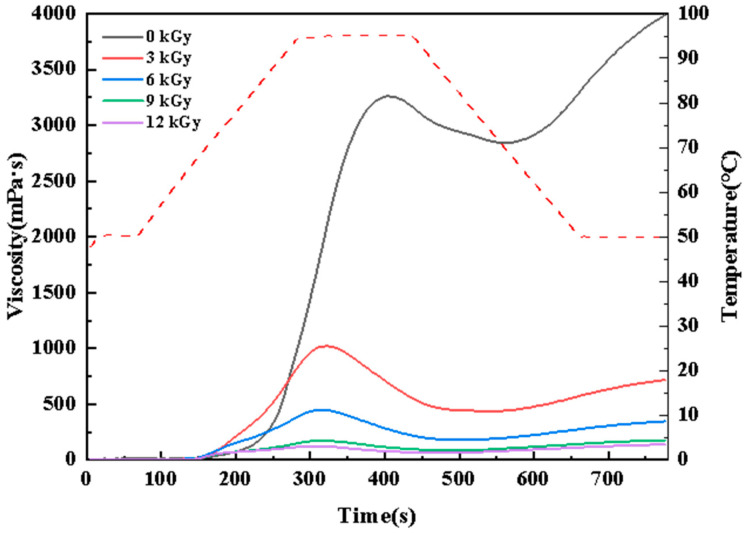
Effect of electron beam irradiation on pasting properties.

As shown in Figure 4 and Table 3, the PV, TV, and FV of the nonirradiated starch were 3184.33 mPa·s, 2768.33 mPa·s, and 3858.67 mPa·s, respectively. The EBI-treated starch exhibited a significant, dose-dependent decrease in PV, TV, and FV decreases (*p* < 0.05), reaching 122.67 mPa·s, 67.33 mPa·s, and 139.33 mPa·s, respectively, at 12 kGy. These declines were attributed to the irradiation-induced disruption, which facilitated granule disintegration under heat and shear, thereby reducing the viscosity retention level and weakening the overall pasting performance [13].

The breakdown (BD), representing thermal paste stability, decreased significantly from 416.00 mPa·s (0 kGy) to 55.33 mPa·s (12 kGy), indicating improved thermal stability after undergoing irradiation. The setback (SB), reflecting short-term retrogradation, decreased from 1090.33 mPa·s to 72.00 mPa·s as the irradiation dose increased, demonstrating that EBI effectively delayed the retrogradation of starch [46].

### 3.5. Pearson Correlation Analysis

**Figure 5:**
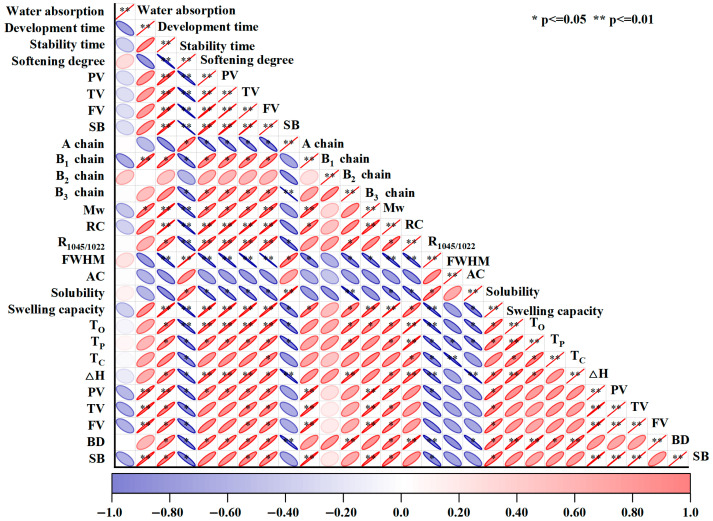
Correlation analysis between starch structural changes and dough quality.

The internal relationships between the starch characteristics and dough quality were investigated via a Pearson correlation analysis. As shown in Figure 5, the dough development time was significantly positively correlated with the proportion of B_1_ chains and starch pasting viscosity (*p* < 0.05), but significantly negatively correlated with the proportion of A chains and FWHM. Similarly, the dough stability time was strongly positively correlated with Mw, RC and swelling (*p* < 0.01), and negatively correlated with the proportion of A chains, FWHM, AC and solubility (*p* < 0.05). The degree of dough softening degree was significantly positively correlated with the A chain proportion, solubility and FWHM (*p* < 0.05), but highly significantly negatively correlated with Mw, RC and the R_1045/1022_ ratio (*p* < 0.01), as well as significantly negatively correlated with the proportions of B_1_ and B_3_ chains, the starch pasting viscosity and thermal properties (*p* < 0.05). Moreover, the dough pasting viscosity were strongly positively correlated with RC, swelling and R_1045/1022_ (*p* < 0.01) and significantly positively correlated with Mw, and the proportions of B_1_ and B_3_ chains, PV and BD (*p* < 0.05). Hence, the key factors affecting the dough quality level were the chain-length distribution, molecular weight, crystalline structure, short-range order, and pasting viscosity of starch during EBI.

## 4. Conclusions

This study elucidated the mechanism by which EBI regulates the quality of wheat dough quality from the perspective of isolated wheat starch. CLSM and SEM revealed that irradiation weakened the interactions between starch and protein, resulting in reduced continuity and compactness of the gluten network. EBI significantly affected the thermomechanical properties of the dough. At 12 kGy, the dough development time, stability time, PV, TV, FV, and SB decreased to 0.970 min, 1.290 min, 1.657 N·m, 1.489 N·m, and 0.926 N·m, respectively. A comprehensive analysis of all the performance indicators indicated that an irradiation dose of 9 kGy resulted in the most pronounced improvement in dough and starch properties, including the optimal amylose content and maximal thermal and rheological performance. A multiscale structural starch characterization scheme revealed that irradiation disrupted the crystalline structure and short-range order, resulting in a pronounced decrease in Mw and an increased proportion of short-chain starch fractions. In addition, compared with nonirradiated starch, irradiated starch exhibited lower PV, TV, and FV values but higher solubility. A correlation analysis further indicated that the molecular weight, crystalline structure, molecular order, and swelling capacity of the starch were the key structural factors governing irradiation-induced changes in dough quality changes. Overall, this study provides not only a theoretical basis for understanding the effects of EBI on the quality of wheat flour from the perspective of the starch structure but also practical guidance: applying approximately 9 kGy of EBI during storage or processing can effectively improve the quality of dough and the functionality of starch. However, considering the national regulations imposed on food irradiation, an appropriate dose should be selected in accordance with the relevant standards, providing a scientific reference for the storage and quality control of wheat flour.

## Figures and Tables

**Table 1:** Effect of electron beam irradiation on the thermomechanical properties of dough.

Dose (kGy)	Water Absorption (%)	Development Time (min)	Stability Time (min)	Softening Degree (N·m)	PV (N·m)	TV (N·m)	FV (N·m)	SB (N·m)
0	52.400 ± 0.000 ^a^	3.920 ± 0.000 ^a^	6.465 ± 0.474 ^a^	0.157 ± 0.001 ^e^	2.173 ± 0.001 ^a^	2.234 ± 0.018 ^a^	3.744 ± 0.054 ^a^	1.510 ± 0.035 ^a^
3	53.700 ± 0.000 ^a^	1.025 ± 0.035 ^b^	4.000 ± 0.000 ^b^	0.289 ± 0.017 ^d^	2.003 ± 0.027 ^b^	1.996 ± 0.028 ^b^	3.302 ± 0.025 ^b^	1.307 ± 0.002 ^b^
6	53.000 ± 0.000 ^a^	0.880 ± 0.000 ^d^	2.690 ± 0.156 ^c^	0.379 ± 0.010 ^c^	1.885 ± 0.006 ^c^	1.781 ± 0.001 ^c^	2.907 ± 0.047 ^c^	1.127 ± 0.047 ^c^
9	53.000 ± 0.000 ^a^	1.000 ± 0.000 ^b c^	1.890 ± 0.014 ^d^	0.468 ± 0.001 ^b^	1.811 ± 0.001 ^d^	1.685 ± 0.006 ^d^	2.697 ± 0.009 ^d^	1.012 ± 0.003 ^d^
12	53.000 ± 0.000 ^a^	0.970 ± 0.000 ^c^	1.290 ± 0.014 ^e^	0.500 ± 0.004 ^a^	1.657 ± 0.009 ^e^	1.489 ± 0.012 ^e^	2.414 ± 0.020 ^e^	0.926 ± 0.008 ^e^

Mean values with different letters are significantly different (*p* < 0.05).

**Table 2:** Effects of electron beam irradiation on molecular weight, relative crystallinity, short-range ordered degree, amylose content, and solubility and swelling.

Dose (kGy)	Mw (g/mol)	RC (%)	R_1045/1022_	FWHM	Amylose Content (mg/g)	Solubility (%)	Swelling (g/g)
0	85,003.53 ± 6.33 ^a^	31.82 ± 1.62 ^a^	0.870 ± 0.030 ^a^	16.49 ± 0.44 ^d^	335.30 ± 5.26 ^c^	7.30 ± 0.13 ^e^	10.29 ± 1.32 ^a^
3	40,118.87 ± 21.29 ^b^	28.97 ± 0.23 ^b^	0.869 ± 0.030 ^a^	17.40 ± 0.01 ^c^	336.95 ± 8.01 ^bc^	12.35 ± 0.22 ^d^	9.18 ± 0.08 ^ab^
6	20,706.36 ± 124.75 ^c^	27.53 ± 0.59 ^bc^	0.837 ± 0.003 ^ab^	18.24 ± 0.18 ^b^	358.45 ± 10.40 ^a^	14.42 ± 0.10 ^c^	8.79 ± 0.19 ^bc^
9	13,148.5 ± 145.98 ^d^	26.72 ± 0.99 ^cd^	0.819 ± 0.023 ^ab^	19.03 ± 0.06 ^a^	373.31 ± 5.27 ^a^	26.00 ± 0.30 ^b^	8.33 ± 0.15 ^bc^
12	8734.38 ± 88.44 ^e^	25.43 ± 1.29 ^d^	0.805 ± 0.002 ^b^	19.31 ± 0.36 ^a^	355.47 ± 5.92 ^ab^	34.75 ± 0.26 ^a^	7.82 ± 0.41 ^c^

Mean values with different letters are significantly different (*p* < 0.05).

**Table 3:** Effects of electron beam irradiation on thermodynamic properties and pasting properties.

Dose (kGy)	T_o_ (°C)	T_p_ (°C)	T_c_ (°C)	ΔH (J/g)	PV (mPa·s)	TV (mPa·s)	FV (mPa·s)	BD (mPa·s)	SB (mPa·s)
0	58.99 ± 0.13 ^a^	63.43 ± 0.21 ^b^	80.16 ± 0.06 ^a^	10.94 ± 0.88 ^a^	3184.33 ± 107.22 ^a^	2768.33 ± 107.37 ^a^	3858.67 ± 157.26 ^a^	416.00 ± 5.57 ^a^	1090.33 ± 54.78 ^a^
3	58.71 ± 0.42 ^a^	63.46 ± 0.18 ^a^	80.12 ± 0.07 ^a^	10.49 ± 0.26 ^ab^	1026.67 ± 12.42 ^b^	631.67 ± 9.29 ^b^	918.33 ± 8.50 ^b^	395.00 ± 11.79 ^b^	286.67 ± 5.86 ^b^
6	57.91 ± 0.15 ^b^	62.66 ± 0.62 ^bc^	79.93 ± 0.20 ^ab^	10.23 ± 0.33 ^ab^	452.67 ± 6.35 ^c^	187.33 ± 4.16 ^c^	351.67 ± 4.04 ^c^	265.33 ± 8.50 ^c^	164.33 ± 5.51 ^c^
9	57.53 ± 0.01 ^bc^	62.14 ± 0.13 ^c^	79.84 ± 0.07 ^b^	9.56 ± 0.27 ^bc^	172.33 ± 10.60 ^d^	86.00 ± 5.20 ^d^	177.33 ± 8.62 ^d^	86.33 ± 6.11 ^d^	91.33 ± 4.16 ^d^
12	57.15 ± 0.20 ^c^	62.06 ± 0.04 ^c^	79.94 ± 0.33 ^ab^	9.00 ± 0.21 ^c^	122.67 ± 2.52 ^d^	67.33 ± 1.53 ^d^	139.33 ± 1.15 ^d^	55.33 ± 3.21 ^e^	72.00 ± 1.73 ^d^

Note: Mean values with different letters are significantly different (*p* < 0.05).

## Data Availability

The original contributions presented in this study are included in the article/Supplementary Material. Further inquiries can be directed to the corresponding authors.

## Supplementary Materials

The following supporting information can be downloaded at: https://www.mdpi.com/article/10.3390/foods15061005/s1, Figure S1: Effect of electron beam irradiation on the thermomechanical properties of dough.
