# Supplementary material for: Electron Beam Irradiation Modulates the Multiscale Structure and Physicochemical Properties of Wheat Starch in Dough Systems
Source: Foods. 2026 Mar 12;15(6):1005. doi: 10.3390/foods15061005 (PMC13025817; doi:10.3390/foods15061005)
Supplement: Supplementary file 1 [file foods-15-01005-s001.zip › Supplementary Figure.pdf]

Supplementary Material

Figure S1

Effect of electron beam irradiation on the thermomechanical properties of dough.

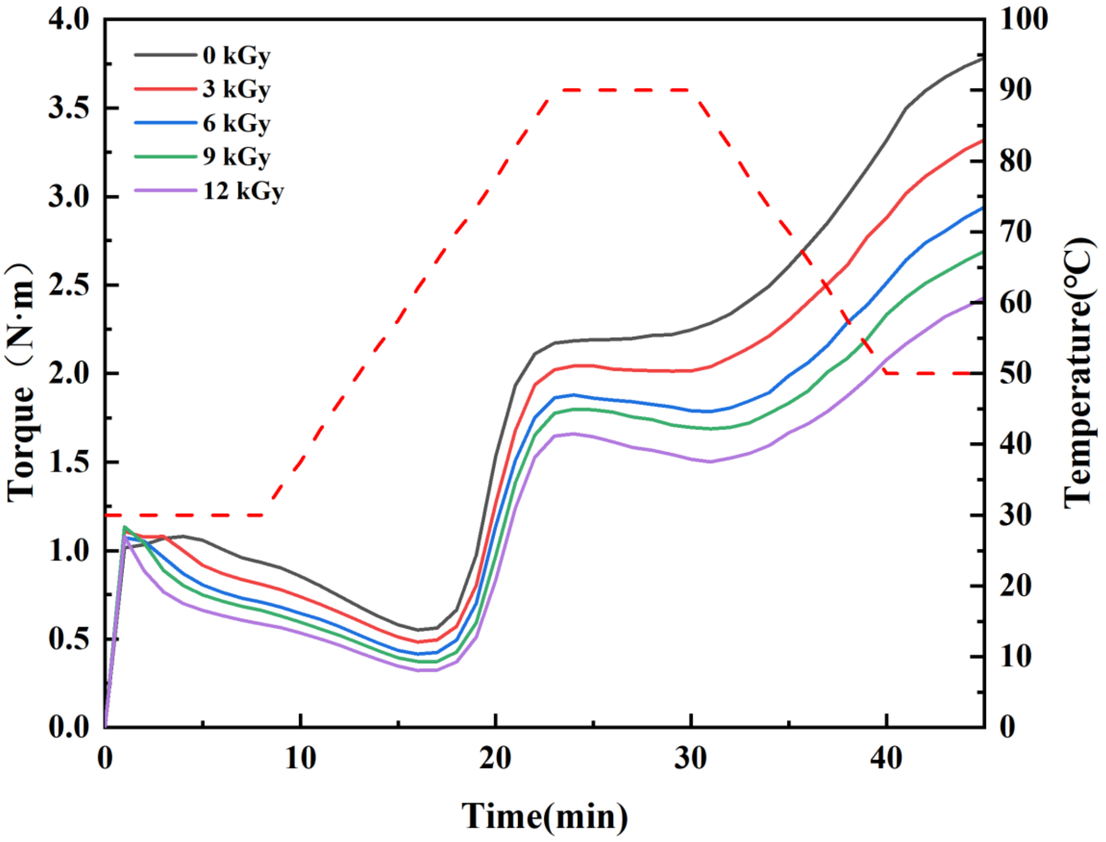
0 kGy
3 kGy
6 kGy
9 kGy
12 kGy
Torque（N·m）
Temperature(°C)
Time(min)
4.0
3.5
3.0
2.5
2.0
1.5
1.0
0.5
0.0
100
90
80
70
60
50
40
30
20
10
0
0
10
20
30
40

Figure S1
